# Evaluation of anticancer effects of carboplatin–gelatin nanoparticles in different sizes synthesized with newly self-assembly method by exposure to IR light

**DOI:** 10.1038/s41598-022-15051-7

**Published:** 2022-06-23

**Authors:** Ferdane Danışman-Kalındemirtaş, İshak Afşin Kariper, Gökçe Erdemir, Esra Sert, Serap Erdem-Kuruca

**Affiliations:** 1 Department of Physiology, Faculty of Medicine, Erzincan Binali Yildirim University, 24100 Erzincan, Turkey; 2 Department of Science Education, Education Faculty, Erciyes University, 38039 Kayseri, Turkey; 3 Aziz Sancar Institute of Experimental Medicine, Department of Molecular Medicine, Istanbul University, 34390 Istanbul, Turkey; 4 Molecular Cancer Research Center (ISUMKAM), Istinye University, 34010 Istanbul, Turkey; 5 Department of Hematology, Istanbul Faculty of Medicine, Istanbul University, 34390 Istanbul, Turkey

**Keywords:** Cancer, Medical research, Chemistry, Materials science

## Abstract

Carboplatin (CP), a platinum analog, is one of the most widely used chemotherapeutic agents in the treatment of colorectal cancer. Although platinum-based drugs are quite effective in anticancer treatments, their use in a wide spectrum and effective treatment possibilities are limited due to their systemic side effects and drug resistance development. In recent years, studies have focused on increasing the therapeutic efficacy of platinum-based drugs with drug delivery systems. Gelatin, a protein, obtained by the hydrolysis of collagen, is a biocompatible and biodegradable material that can be used in nano drug delivery systems. In this study, CP-loaded gelatin-based NPs (CP-NPs) were exposed to IR light in different temperatures at 30, 35, 40, 45, and 50 °C and characterized by FESEM-EDX, FTIR, UV–Vis, DLS. Accordingly, we synthesized gelatin-based CP-NPs of different sizes between 10–290 nm by exposure to IR. We found that CP-NPs-50, 16 nm nano-sized, obtained at 50 °C had the most cytotoxicity and was 2.2 times more effective than the free drug in HCT 116 colon cancer cells. Moreover, we showed that the cytotoxicity of CP-NPs-50 in normal HUVEC cells was lower. Additionally, we demonstrated that CP-NPs enhanced apoptotic activity while not developing MDR1-related resistance in colon cancer cells. In this study, for the first time drug loaded gelatin-based nanoparticles were synthesized in different sizes with a newly self-assembly method by exposing them to infrared light at different temperatures and their anticancer effects were evaluated subsequently.

## Introduction

Colorectal cancer is the second most common cause of mortality from cancer among both men and women^1^. Despite various treatment options, it continues to be an important health problem worldwide. Although the clinical applications of platinum-based drugs are extremely effective, their toxicity profile restricts their extensive and effective application. Therefore, it is extremely important to develop new chemotherapy drug formulations that are more effective in cancer cells by reducing systemic side effects and drug resistance in the organism. The recent studies focus on developing new platinum-based drug formulations, expanding the therapeutic aspect. Carboplatin (CP), a platinum analog, is one of the most widely used chemotherapeutic agent in the treatment of colorectal cancer. CP was approved by the FDA in the 1980s and continues to be used in the treatment of various cancers. It has a broad spectrum of chemotherapy in various malignancies including ovarian cancer, small cell lung cancer, head and neck cancer, thoracic cancers, and bladder cancer^2^. Although CP, a derivative of cisplatin, has a similar therapeutic action, it differs in structure and toxicity from cisplatin. CP can create defects in DNA through platinum and cause cell death by inhibiting replication transcription. However, its side effects and development of drug resistance can significantly limit the full potential scope of the drug and cause serious problems^3^. Its common side effects such as nausea, vomiting, nephrotoxicity, neurotoxicity, and ototoxicity limit its use^4^. Additionally, carboplatin cannot be used at an effective dose due to the development of drug resistance such as cisplatin^5, 6^. Therefore, it is imperative to develop new formulations in order to benefit from its therapeutic effect with the highest efficacy.

Gelatin is all-purposed biopolymer and is widely utilized in cosmetic, food, pharmaceutical, and medical area. Gelatin is formed by the hydrolysis of collagen, during which the collagen is denatured and its triple helix structure loosens and loses its secondary structure. Since collagen is insoluble in water, it hydrolyses to gelatin in acidic or basic solutions. During gelling, the chains of gelatin undergo a conformational rearrangement and the triple-helix structure gets partially renewed. Accordingly, two sort of gelatin are produced: Type A by acid hydrolysis and Type B by base hydrolysis^7, 8^. In addition, gelatin has two different isoelectric points, such as type A and type B. While the isoelectric point of type A is between 7–9, the isoelectric point of type B is around 4–5. Therefore, different types of nanoparticles can be obtained from the nano-sized gelatins to be produced^9, 10^. Being able to control the physical form of the gelatin molecule with temperature also increases the possibility of production at nanoscale.

Gelatin nanoparticles are natural polymers and widely used as drug carriers to target tumor tissues in diseases such as cancer due to their biocompatibility and biodegradability^11^. Furthermore, the major advantages of gelatin nanoparticles is not only very low toxicity, but also them having the opportunity for multiple modifications with excellent pharmaceutical excipient, thermos-reversible gel formation, high purity, low immunological and non-allergenic properties, steerable physical parameters. Moreover, it can also be produced easily at low cost and integrates easily with many molecules, and the fact that the gelatin matrix molecule has amino acid side chains allows for the formation of numerous other modifications. The surface of gelatin nanoparticles can be modified with site-specific ligands, cationized with amine derivatives, or coated with polyethyl glycols to achieve targeted and sustained release drug delivery. Compared with other colloidal carriers, gelatin nanoparticles are more stable in biological fluids to provide the desired controlled and sustained release of entrapped drug molecules^7, 10^. Accordingly, gelatin is a unique nanocarrier to reduce the systemic side effects of carboplatin and increase its accumulation in cancer cells, including improved efficacy to overcome drug resistance. Although there are some studies on gelatin-based nanodrugs, which are usually related to paclitaxel^12–18^, there are few studies with the combination of carboplatin gelatin.Their nanoparticle size is very large and their in vitro anticancer activity has not been investigated^19, 20^.

In order to produce nano gelatin, the literature so far has included (1) two-step desolvation^21, 22^ (includes centrifugation and lyophilization steps by adding crosslinker), (2) simple coacervation^23, 24^ (utilizing liquid–liquid extraction), (3) solvent evaporation^25, 26^ (evaporation of the solvent can also cause uneven particle size distribution in NP production), (4) microemulsion^27, 28^ (emulsification with surfactants). It is difficult to control the size with these methods except for microemulsion. In addition to all these, no other method that can provide self-assembly and size control while loading drugs into the carrier has been found in the literature. Therefore, in this study, we examined whether we can control the size of a macromolecule such as gelatin, whose physical form changes with temperature, by an IR light source.

In this study, for the first time in the literature, a drug (CP) was adsorbed to macromolecule gelatin induced by an IR light source and its anticancer effects were investigated. The originality of this work is not only to create a drug delivery system by stimulating it with an IR light source, but also to add carboplatin to this system. A literature review reveals that paclitaxel is used for bonding to gelatin-based drug carriers and there are very few studies on the combination of carboplatin and gelatin. As far as we know, there is no study in the literature realizing direct (self-assembled) gelatin macromolecule-nanodrug synthesis in different nanoscales when stimulated at different temperatures and investigating their anticancer effects. Obtaining nanoparticles with this method allows the drug to self-attach to the macromolecule, thus saving time, cost and effort. We believe that such a drug storage method will contribute to both nanotechnology and effective anticancer drug studies.

## Materials and methods

Colon cancer cells (HCT116) and human umbilical vein endothelial cells (HUVEC) were obtained from the American Type Culture Collection (ATCC). As anticancer drug Carboplatin was purchased from Koçak Farma and Gelatin A from Sigma (USA). Regarding chemicals, 3-(4,5-Dimethylthiazol-2-yl)-2,5-diphenyltetrazolium bromide kit (MTT), Dimethylsulfoxide (DMSO), Dulbecco's Modified Eagle's Medium (DMEM) for cell culture were obtained from Sigma (St. Louis, USA) Trypsin, penicillin, streptomycin from Gibco (UK). Phosphate buffer saline (PBS) tampon solution for release studies was acquired from Thermo Fisher (USA). AnnexinV/PI kits were procured from Becman Coulter (USA) while Anti-MDR1/ABCB1 Antibody (UIC2) for P-gp evaluation was obtained from Santa Cruz (USA).

### Preparation of nanoparticles

**Figure 1:**
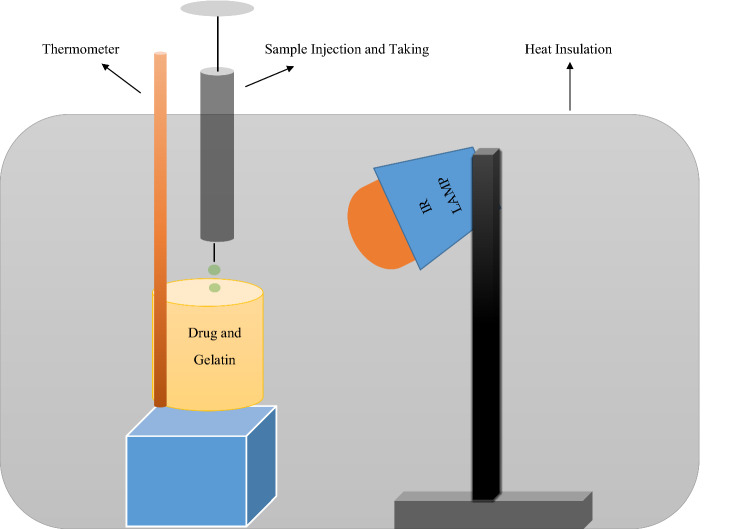
Schematic representation for preparation of CP loaded gelatin-based NPs by IR.

As seen in Fig. 1, a setup was prepared to obtain carboplatin loaded gelatin-based nanoparticles. 20 mL of carboplatin (1 mg/mL, distilled water) and 20 mL of gelatin (10 mg/mL, distilled water) stock solutions were used. A 150-W IR light source (Rotlichtlambe IR 150) was placed approximately 20 cm from the sample and at a 45° angle to illuminate the entire sample (Fig. 1) and a thermometer was placed inside the sample container. Thus, self-assembly binding of the CP to the gelatin nano carrier was expected by exposure to IR light and vibrations. As the temperature of the solution in the sample container reached 30–35–40–45–50 °C, 2 mL of solution was taken at each specified temperature with a separate injector immersed in the sample container. After the final sampling at 50 °C, there was about 20 mL more of the mixture in the sample container. Although the protein structure of gelatin is denatured above 40 °C, it preserves its protein properties, but at temperatures above 50 °C, the protein structure may degrade and lose its protein properties. For this reason, we did not include temperatures above 50 °C into our study^8, 29^. In addition, since the room temperature was also approximately 24 °C, it was appropriate to start taking samples of gelatin-based NPs at 30 °C. The experimental setup was protected with foams providing thermal insulation as shown in the figure.

### Particle size distribution and zeta potential distribution analysis

The size distribution of the gelatin based CP-NPs was determined by dynamic light scattering (DLS) analysis by Zetasizer Nano ZS (4 mW He–Ne laser operating) at room temperature., distilled water was used as reference liquid.

### Fourier transform infrared spectroscopy analysis

Fourier Transform Infrared spectroscopy (FTIR) analysis were performed using the BRUKER ALPHA spectrometer with diffuse reflection mode at 4 cm^−1^ resolution. The measurements of each sample were recorded after 10 scans. Before the measurements, the correction was made by taking pure water as a reference.

### EDX-FESEM analysis

First, each sample were individually dropped onto an amorphous glass lamella. The samples were then allowed to dry at room temperature under normal atmospheric conditions and the dried samples were used for FESEM-EDX analysis. Next, the surface characteristics of the gelatin-based CP-NPs were examined by a Gemini 500 digital transmission electron microscope (SEM). Quantitative elemental analyzes of each CP-NPs were determined by an EDX spectrometer attached to SEM.

### UV–Vis spectroscopy

Absorbance measurements of the gelatin based CP-NPs were evaluated by Hach Lange 500 Spectrophotometer at 25 °C and distilled water was used on the reference beam. The absorbance spectra of the gelatin-based CP-NPs were measured in the range of 200–1100 nm wavelength.

### Drug release studies

To evaluate drug release profile of drug loaded gelatin based NPs, CP from gelatin based NPs were used in a dialysis membrane in pH 7.4 PBS tampon buffer, at 37 °C. Wavelengths of the carboplatin was measured at 235 nm^30^ using the UV spectrophotometer. Then, 2 mL of gelatin based carboplatin loaded NPs were put in a dialysis membrane and placed in 50 mL of buffer solutions of pH 7.4 at 37 °C. Then, the sample was shaken for different periods and samples were taken at different times and measurements of the samples were taken in a UV spectrometer at 235 nm.

### Cell culture

HCT116 colon cancer and HUVEC normal cell lines purchased were cultured in DMEM supplemented with 10% FBS, 100 µg/mL streptomycin and 100 units/mL penicillin in 5% CO_2_ atmosphere in a humidified incubator. To procure enough cells for the cell studies, the medium of cells was changed every 2–3 days and passaged using trypsin when the cell confluent reached 70–80% confluence in the flask.

#### Cytotoxicity evaluation by MTT test

The cytotoxic effects of free CP and CP-NPs-30, CP-NPs-35, CP-NPs-40, CP-NPs-45, CP-NPs-50 separately determined using 3-(4,5-dimethylthiazolyl)2,5-diphenyl-tetrazolium bromide (MTT) assay on HCT116 cancer and HUVEC normal cells. Firstly, the cells were cultured in 96-well plates with approximately 1 × 10^5^ (cells/1 mL) in each well as 90 μL. Later, different concentrations of CP and CP-NPs-30, CP-NPs-35, CP-NPs-40, CP-NPs-45, CP-NPs-50 compounds were prepared with DMEM. Then, the compounds were treated in range of 2–100 μM concentration as total 10 μL to each well. The cells treated by the compounds were incubated for 72 h. Subsequently, MTT solution (10 μL of 5 mg/mL PBS) were added to each well and 4 h later 100 μL DMSO solution was added to turn formazan crystals formed by MTT solution^31^. The absorbances were measured by an ELISA microplate reader (AllshengFlexa200) at 570 nm. The all experiments were repeated three times at least.

### Apoptosis/necrosis determination

Apoptosis and necrosis were assessed by flow cytometry after 72 h treatment at IC_50_ concentrations of CP and CP-NPs-50 compounds (CP: 87 µM, CP-NPs-50: 39.39 µM) on HCT116 cells. HCT116 colon cancer cells were cultured as 2 × 10^5^ cells with 1 mL DMEM in 6-well plate on each well. Afterwards, IC_50_ concentrations of CP and CP-NPs-50 compounds were seeded to 6-well plate. After 72 h incubation period, the cells were taken into falcons and medium was removed after centrifugation for 10 min. Subsequently 1 mL PBS was put to the cells and centrifuged. This process was repeated 3 times.

### P-glycoprotein (Pgp) determination by anti-MDR1/ABCB1 antibody

HCT116 cell lines were incubated with CP and CP-NPs-50 at IC_50_ concentrations at 2 × 10^5^ cells/mL. 10 µL of Anti-MDR1/ABCB1 Antibody (UIC2) was used. Pgp activation of HCT116 cells was measured by flow cytometry after an incubation period of 15 min.

## Results and discussion

**Figure 2:**
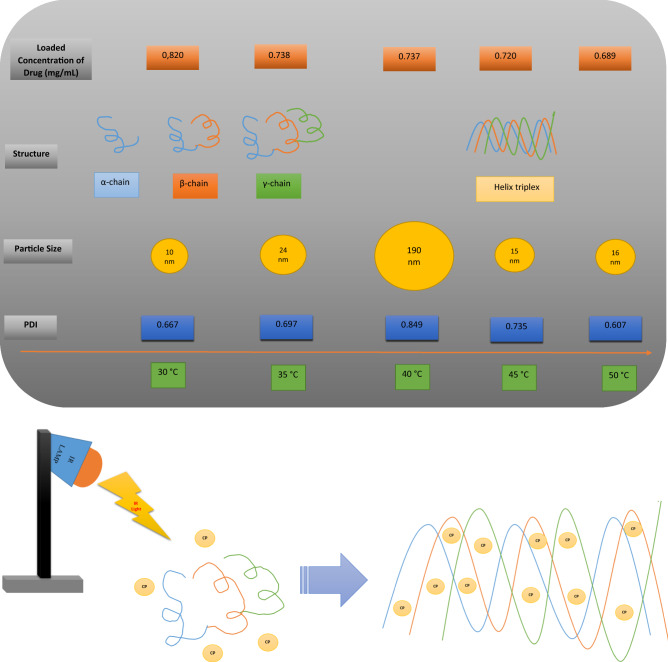
Comparative schema of structure, particle size, PDI value and loaded concentration of different gelatin-based CP-NPs exposed to IR light at different temperatures (30, 35, 40, 45, 50 °C).

In this study, IR light with 150-W energy was used as a thermal and vibrational energy source to synthesize gelatine based CP-NPs in different sizes. First, 10 nm and 24 nm gelatin-based CP-NPs were obtained at 30 and 35 °C respectively, and afterwards, interestingly, the NPs sizes increased to 190 nm at 40 °C, and, then the size of gelatin-based CP-NPs decreased to 15 nm and 16 nm at 45 and 50 °C respectively (Fig. 2). According to Fig. 2, gelatin-based CP-NPs of different characters and sizes are formed at different temperatures when exposed to heat via IR light. CP-NPs-50 synthesized at 50 °C were more stable than the others (PDI = 0.607). These were also found to have the highest cytotoxicity on HCT116 colon cancer cells. CP-NPs-40, which was synthesized at 40 °C and had the largest nanoparticle size at 190 nm, had the highest PDI value of 0.849 and was least effective in HCT 116 colon cancer cells (Supplementary Fig. 1, Figs. 2, 6).

According to Fig. 2, while 0.82 µg/mL CP was loaded on the gelatin nano-carrier at 30 °C, 0.738, 0.737, 0720 and 0.689 µg/mL CPs were loaded at 35, 40, 45 and 50 °C, respectively. Accordingly, as the temperature increased, the amount of drug that the gelatin nano-carrier could carry decreased (Supplementary Fig. 2 and Supplementary Table 1). At the same time, EDX analysis also shows the drug concentration by the relative % of platinum within (Supplementary Fig. 3). Accordingly, the amount of platinum in the structure of gelatin-bound CP-NPs was: Pt atomic 0.50% at 30 °C, Pt atomic 0.43% at 35 °C, Pt atomic 0.40% at 40 °C, Pt atomic 0.36% and 50% at 45 °C, Pt atomic was measured as 0.22% at 50 °C. These drug ratios were in good correlation with the results obtained from the UV–Vis spectrum.

In this study, the reason for the gelatin-based NPs we synthesized, having different NP sizes with the variable thermal effect of the IR lamp, is that gelatin has a thermo-reversible gel formation. It is reported that gelatin forms a tight gel at 26–30 °C, starts to melt after 33–34 °C and reaches a solubility above 40 °C^7^. In this study, gelatin-based NPs were in tight gel form at 30 °C, holding less water and having a size of 10 nm, reaching 16 nm at 35 °C, again a small size, while at 40 °C, it had the largest size of 190 nm with the highest water content. Interestingly, with increasing solubility of gelatin at 45 and 50 °C, it is noteworthy that again 15 and 16 nm small size NPs were obtained. It is known that gelatin has a high water holding capacity at 40 °C^32^. The large nanoparticle size (190 nm) of CP-NPs-40 synthesized at 40 °C in our study is supported by the literature^7^. It is clearly seen that gelatin-based CP-NPs of different characters and sizes are formed when heated to different temperatures via IR Light.

**Figure 3:**
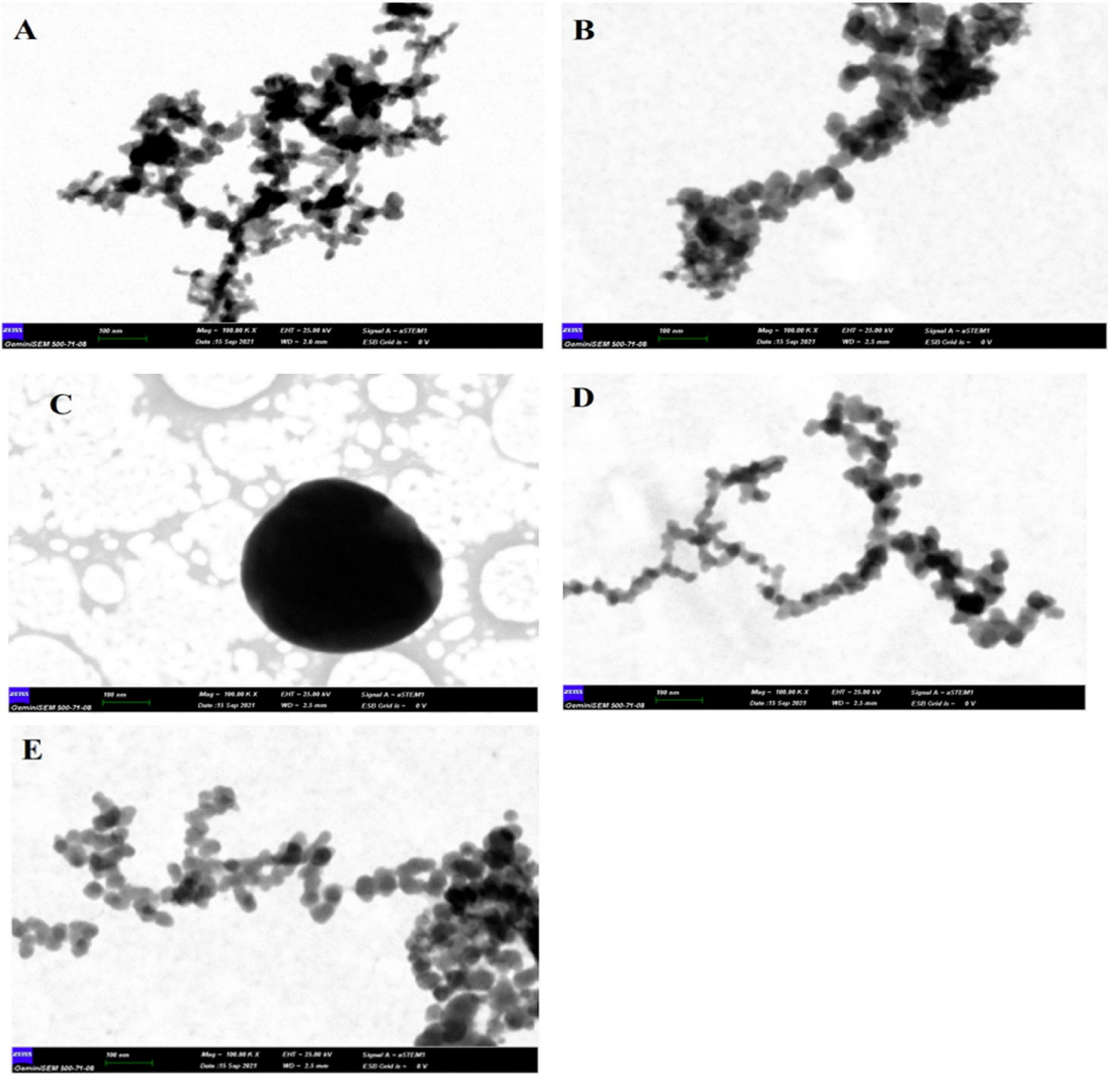
FESEM images of CP-NPs. (**A**) 30 °C, (**B**) 35 °C, (**C**) 40 °C, (**D**) 45 °C, (**E**) 50 °C.

As seen in Fig. 3, the FESEM images are in good correlation with the nanosize measurements of DLS (Supplementary Fig. 2). According to the DLS results, the nanosizes of the gelatin-based CP-NPs obtained at 30 °C were between 7–15 nm, while they ranged between 15–32 nm at 35 °C and 141–255 nm at 40 °C, 11–24 nm at 45 °C and 11–24 nm at 50 °C. According to the FESEM images, after each sample was dried on the substrate, nanoparticles of exactly these sizes were detected very clearly, except for some agglomeration.

**Figure 4:**
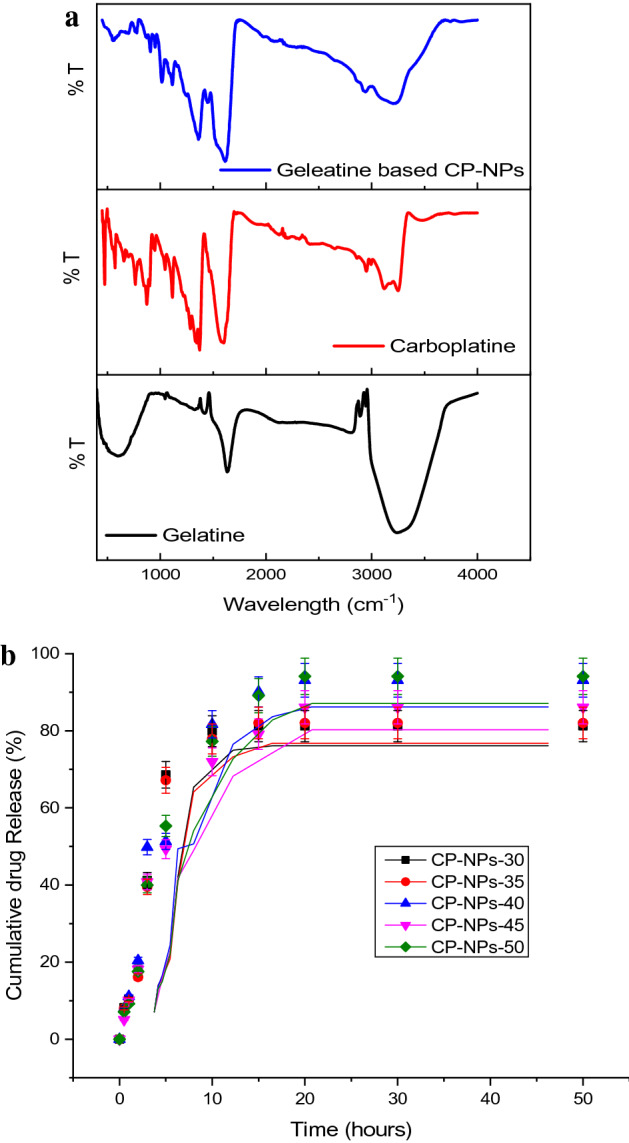
(**A**) FTIR Spectrum of Gelatine, Carboplatine and Gelatine based CP-NPs. (**B**) Drug release rate (%) of CP-NPs-30, CP-NPs-35, CP-NPs-40, CP-NPs-45, CP-NPs-50 compounds.

Figure 4A shows the infrared spectra of gelatin (Gel), free carboplatin (CP) and Gelatin-based CP-NPs (CP-NPs). Considering the IR spectrum of gelatin, vibration signals of 3263 cm^−1^ and 1637 cm^−1^, respectively, originate from amide-I and amide-II bands. The reason why the peak at 3263 cm^−1^ is wide and flat in this way is due to the water in the environment. Amide-I, C=C bond stretch of amide proteins; amide II, N–H vibrational groups and stretching vibrations of C–N groups have contributed to these bands^33^. When we look at the IR spectrum of free carboplatin, the –NH vibration signals of the NH3 group bound to the platinum ligand at 3263–3123 cm^−1^, the –CO and –C=O vibrational signals of the ester group at 1364 cm^−1^ and 1602 cm^−1^, 2964 at cm^−1^, –CH vibration signals in the ring structure in carboplatin were detected^34^. Complex vibration signals in the fingerprint region were not taken into account. Since the IR spectra of the gelatin-based CP-NPs we obtained at different temperatures were almost the identical and the spectrum of one of the CP-NPs samples is given as an example. According to the IR spectrum of gelatin-based CP-NPs, it is seen that some specific peaks of drug and macromolecule gelatin such as –CH aliphatic and N–H, C–N were shifted. In fact, some coalescence in the amide-I and amide-II bands is also striking. This spectrum is a serious proof that free carboplatin is adsorbed by the gelatin macromolecule. This proof is also supported by UV–Vis spectra and EDX analyses.

**Figure 5:**
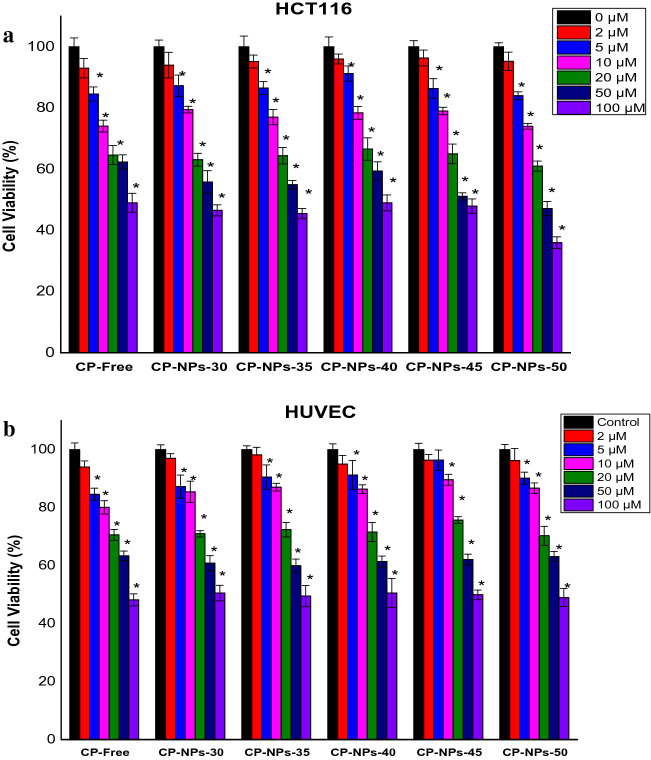
(**A**) The cell viability (%) of free CP and CP-NPs-30, CP-NPs-35, CP-NPs-40, CP-NPs-45, CP-NPs-50 compounds on HCT116 colon cancer cells. Data are shown as mean ± SD after 3 independent experiments. p-value was calculated according to control by Student's *t* test using graph pad (* indicates p-values < 0.005). *CP* Carboplatin. (**B**) The cell viability (%) of free CP and CP-NPs-30, CP-NPs-35, CP-NPs-40, CP-NPs-45, CP-NPs-50 compounds on HUVEC normal cells. Data are shown as mean ± SD after 3 independent experiments. p-value was calculated according to control by Student's *t* test using graph pad (* indicates p-values < 0.005). *CP* Carboplatin.

Cumulative drug release of gelatin-based CP-NPs synthesized at different temperatures was monitored for 50 h at physiological pH (pH 7.4) and 37 ± 0.5 °C. According to Fig. 5, 94% CP was released from CP-NPs-50 after 20 h, 93% CP was released from CPNPs-40 at the end of 20 h, and 81% from CP-NPs-30. According to Fig. 4B, it is seen that CP-NPs-50 has the highest release and CP-NPs-30 has the lowest release.

Table 1 and Fig. 5A show the cytotoxic effects of free CP and gelatin-loaded CP-NPs on HCT-116 colon cancer. According to Table 1, the IC_50_ value of free CP on HCT-116 cells was 87.75, while CP-NPs-35, CP-NPs-40, CP-NPs-45 and CP-NPs-50 were 62.25, 60.45, 78.01, 60.21, 39.39 µM respectively. Accordingly, all CP-NPs were more effective than free CP on HCT116 cells. Moreover, gelatin-based CP-NPs-50 was by far the most effective in all samples. Moreover, it is noteworthy that the CP-NPs-50 (IC_50_: 39.39 µM) we synthesized is 2.22 times more cytotoxic than free CP on cancer cells. Furthermore, regarding normal HUVEC cells (Fig. 5B), the IC_50_ values of all CP-NPs above 80 µM indicate that the nanoparticles we synthesize are specific to cancer cells. Thus, after treatment with CP-NPs, colon cancer cells would be killed with 2.22 times lower concentration of CP, while side effects would be significantly reduced. Our findings show that gelatin-based NP, which we synthesized at 50 °C (CP-NPs-50), is a promising agent for colon cancer treatment.

**Table 1:** The IC_50_ values (µM) of CP and gelatine based CP-NPs. Significant values are in bold.

Compounds	The IC_50_ values (µM) of CP and gelatine based CP-NPs	
	HCT116	HUVEC
CP-Free	**87.75**	**107.83**
CP-NPs-30	62.25	92.25
CP-NPs-35	60.45	85.10
CP-NPs-40	78.01	93.76
CP-NPs-45	60.21	83.88
Cp-NPs-50	**39.39**	**84.28**

Jahanshahi et al. synthesized the gelatin nanoparticles at temperatures of 40, 50, 55, and 60 °C and when they compared the NPs, the optimum temperature for gelatin nanoparticles was 50 °C^35^. In another study, Jahanshahi et al. suggested that it is not possible to form nanoparticles at low temperature due to the highly viscous nature of gelatin, and that the particle size increases above 50 °C. This situation is thought to be related to the gelation property of the gelatin. When the viscosity decreases and the temperature increases, the triple helix structure dissolves at 50 °C^35, 36^. Although our study also supports that 50 °C is the best temperature for gelatin nanoparticles, the anticancer effects of drug–gelatin-based nanoparticles produced at different temperatures were compared for the first time in this study. In addition, in this study, small-sized nanoparticles (CP-NPs-30, CP-NPs-35) were obtained which can be loaded with more drugs at low temperatures. However, their drug release and cytotoxic effect are not better than CP-NPs-50.

**Figure 6:**
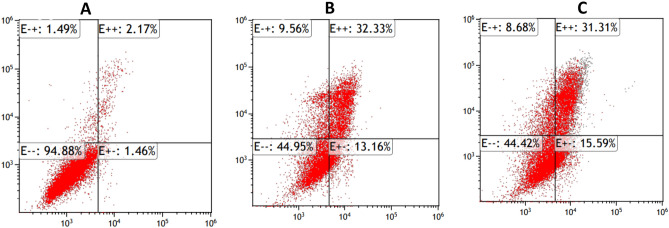
Annexin V-FITC/PI staining for evaluating apoptosis. HCT116 colon cancer cells were treated with free CP (**B**), CP-NPs-50 (**C**) at IC50 concentration for 72 h and compared with untreated cells (**A**). Apoptosis–necrosis ratio was determined by flow cytometry.

To determine apoptotic activity (in Fig. 6), among gelatin-based CP-NPs, CP-NPs-50, which are the most effective in cancer cells were chosen and compared with free CP by flow cytometry analysis. Apoptotic activity was assessed using the concentration at IC_50_ values of the compounds to match with cytotoxicity tests after 72 h. That is, a concentration of 39.39 µM of CP-NPs-50 was applied against 87.75 µM free CP. Free carboplatin caused 13.16% early apoptosis, CP-NPs-50 caused 15.59% early apoptosis, 32.33% late apoptosis and 9.56% necrosis were observed with CP, while 31.31% late apoptosis and 8.68% necrosis were observed with CP-NPs-50. Accordingly, it was determined that cell death by apoptosis occurred at a concentration less than half of CP-NPs-50 compared to free CP. According to these results, it is seen that more effective cancer cell death can be achieved with a lower CP concentration as a result of the accumulation of CP-NPs-50 in cells with the effect of CP-NPs on cancer cells. As a result of our findings, it is clear that CP-NPs-50 increases apoptotic activity at lower concentrations compared to free CP.

**Figure 7:**
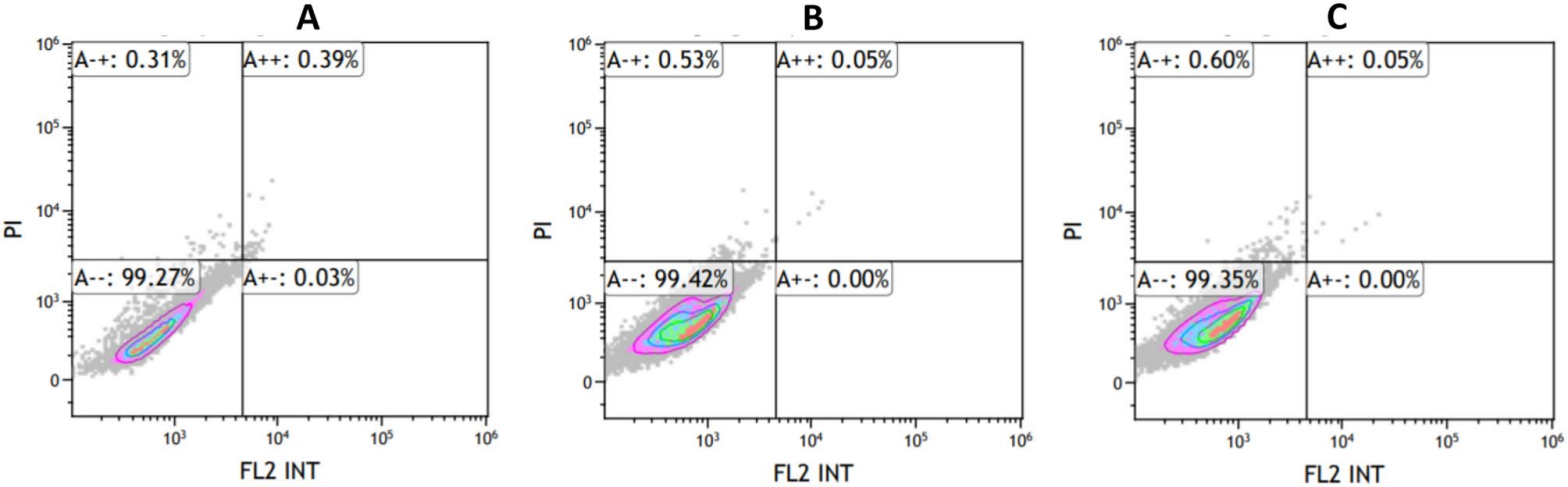
P-gp expression (MDR1) on the HCT116 cells. (**A**) Control, (**B**) Carboplatin, (**C**) CP-NPs-50.

The effects of CP and CP-NPs-50 on drug resistance were evaluated in HCT116 cells by flow cytometry analysis by measuring Pgp expression (MDR1). Cells treated with CP and CP-NPs were compared with untreated cells for MDR1 activity. According to MDR1 measurements, free CP and CP-NPs-50 did not change Pgp expression in drug treated cells compared to untreated control cells (Fig. 7). This indicates that the nanoparticles did not develop MDR1-related resistance in colon cancer cells and can be safely used in therapy.

While CP-NPs-50 had the highest cytotoxic effect in cancer cells, CP-NPs-40 had the lowest toxicity on cancer cells. It is not a coincidence that CP-NPs-50 is around 16 nm and CPNPs-40 is around 190 nm, since it is the size of the NPs that significantly influences cytotoxicity.

It has been reported that the particle size, shape, surface charge and chemistry, stability of nanoparticles seriously affect the contact of NPs with the cell surface and signal transmission^37^. It is also notable that CP-NPs-40 has the highest PDI, while CP-NPs-50 has the lowest PDI (Fig. 2). This also shows that CP-NPs-50 is more stable.

Our findings show that gelatin-based NP, which we synthesized at 50 °C (CP-NPs-50), is a promising agent for colon cancer treatment.

## Conclusion

For the first time in the literature, drug-macromolecule complexes in different nanosizes at different temperatures were successfully produced by the self-assembly method by means of an IR light source and were investigated for their anticancer activity. With this method, which we proved in our study, we achieved to produce different nano-sized drug-macromolecule complexes only by temperature difference via IR lamp. It is also important that the gelatin we choose as the drug delivery system is a cheap, biocompatible, and biodegradable material. This method also will reduce time and cost in production, both for researchers and also in terms of mass production. Subsequently, anticancer studies of the compounds synthesized in the study were carried out. It has been observed that all CP-NPs were more effective than free CP on HCT116 cancer cells. Moreover, gelatin-based CP-NPs-50 was by far the most effective in all samples. Moreover, it is noteworthy that the CP-NPs-50 (IC_50_: 39.39 µM) we synthesized is 2.22 times more cytotoxic than free CP on cancer cells. CP-NPs-50 was selective to cancer cells when compared with normal HUVEC cells. In addition, it would be a serious therapeutic advantage for a drug such as carboplatin, which has very high side effects, to be effective at 2.2 times lower concentration. Our findings show that gelatin-based NP, which we synthesized at 50 °C (CP-NPs-50), is a promising agent for colon cancer treatment.

Therefore, according to the results of the study, it is understood that this drug storage method will make a serious contribution to both nano technology and drug studies.

## Supplementary Information

Supplementary Information.

## Data Availability

The data of the article is available in public.
