# Supplementary material for: Evaluation of anticancer effects of carboplatin–gelatin nanoparticles in different sizes synthesized with newly self-assembly method by exposure to IR light
Source: Sci Rep. 2022 Jun 23;12:10686. doi: 10.1038/s41598-022-15051-7 (PMC9226150; doi:10.1038/s41598-022-15051-7)
Supplement: Supplementary file 1 — Supplementary Information. [file 41598_2022_15051_MOESM1_ESM.docx]

**Supplementary Materials for:**

**Evaluation of Anticancer Effects of Carboplatin-Gelatin Nanoparticles in Different Sizes synthesized with Newly Self-Assembly Method by Exposure to IR Light**

Ferdane Danışman-Kalındemirtaş^a^, İ. Afşin Kariper^b^^[[1]](#footnote-1)^, Gökçe Erdemir^c,d^, Esra Sert^e^, Serap Erdem-Kuruca^e^

*^a^Erzincan Binali Yildirim University, Faculty of Medicine, Department of Physiology, Erzincan 24100, Turkey*

*^b^Erciyes University, Education Faculty, Department of Science Education, Kayseri 38039, Turkey*

*^c^Istanbul University, Department of Molecular Medicine, Aziz Sancar Institute of Experimental Medicine, Istanbul 34390, Turkey*

*^d^Istinye University, Molecular Cancer Research Center (ISUMKAM), Istanbul 34010, Turkey*

*^e^Istanbul University, Istanbul Faculty of Medicine, Department of Hematology, Istanbul 34390, Turkey*

Number of pages: 10

Number of figures: 10

Number of tables: 1

***Corresponding author: İ. Afşin Kariper**

**e-mail:** akariper@gmail.com

**DLS Analysis Results**


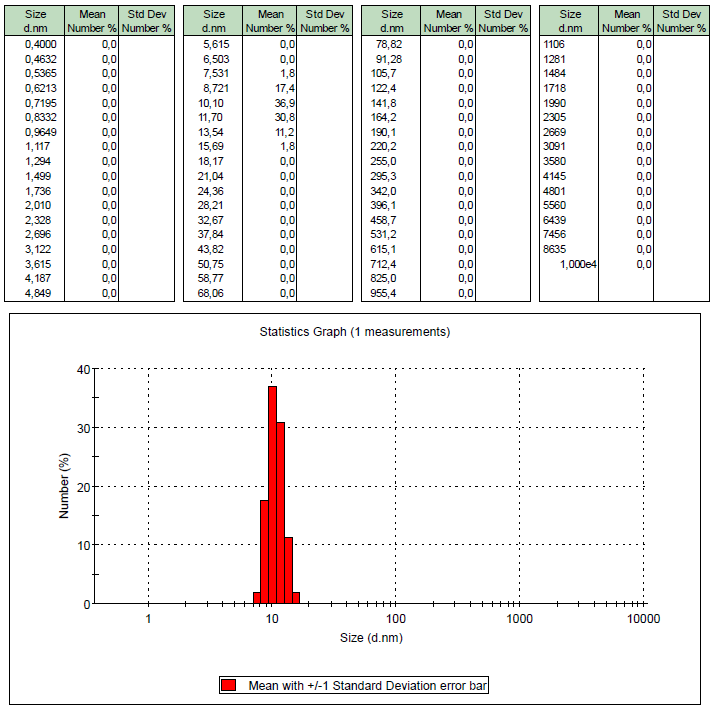


**Supplementary Figure 1 (a) 30 °C**


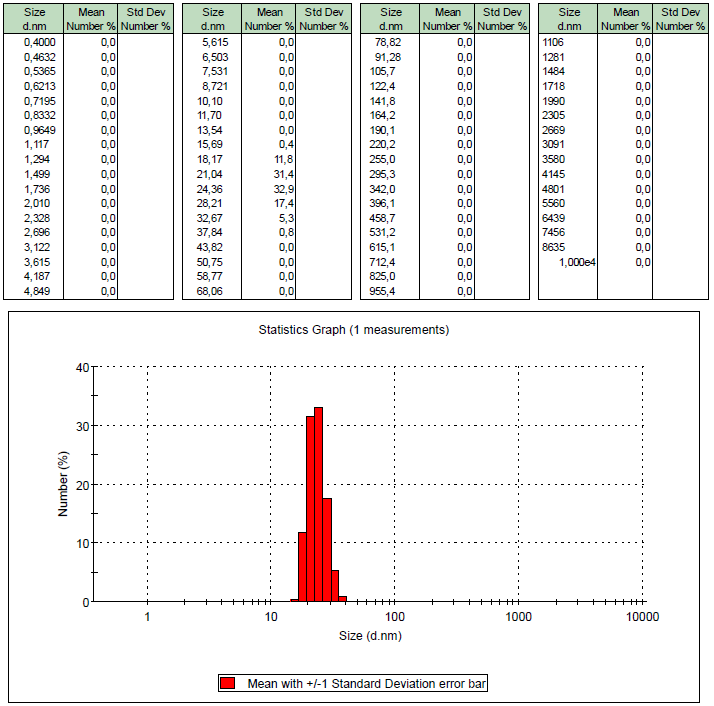


**Supplementary Figure 1 (b) 35 °C**


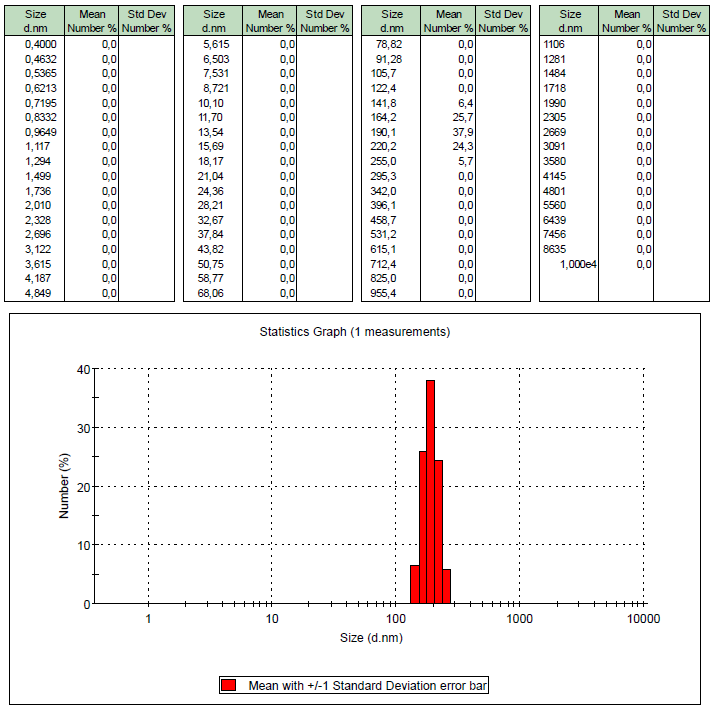


**Supplementary Figure 1 (c) 40 °C**


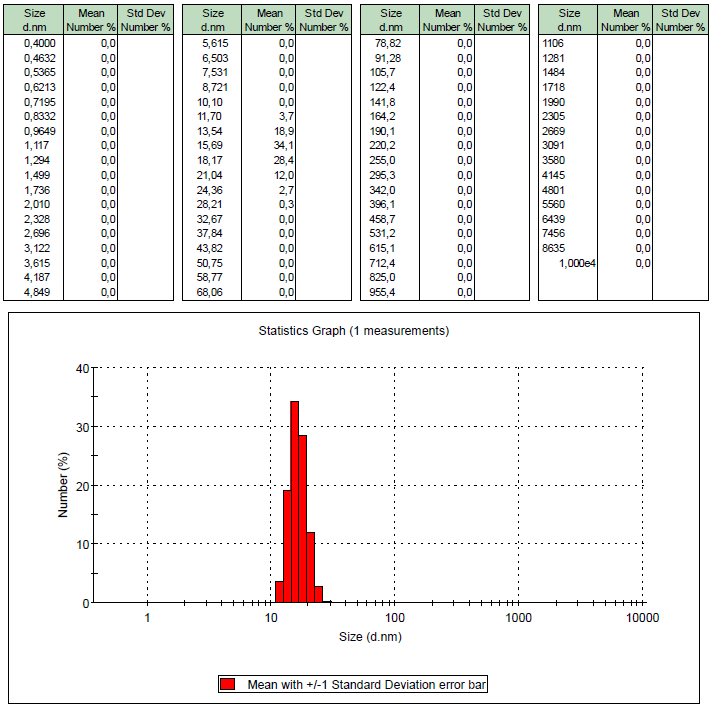


**Supplementary Figure 1 (d) 45 °C**


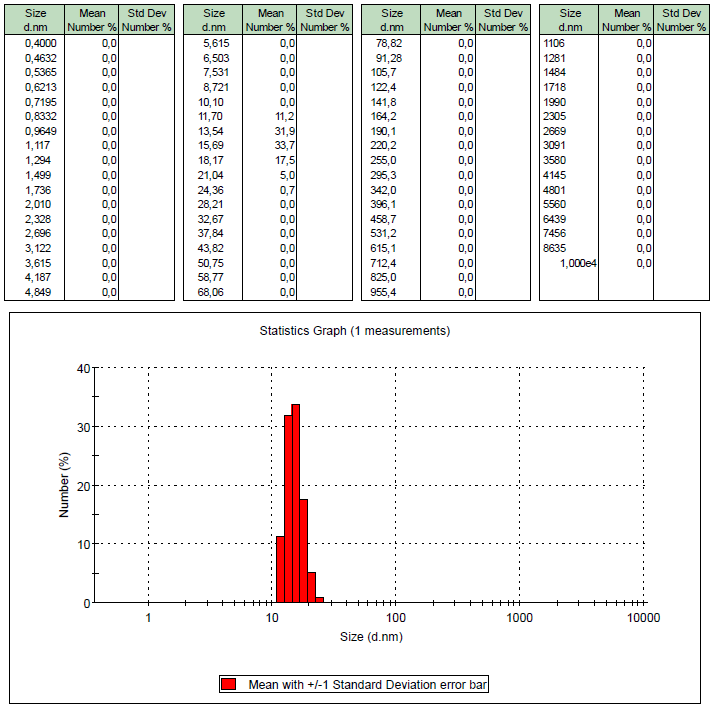


**Supplementary Figure 1 (e) 50 °C**

**UV-VIS. Spectrum**

**Supplementary Figure 2**

For carboplatin in aqueous medium, the maximum absorbance was observed at 317 nm in its presence alone. As the sample was taken from the experimental setup, the absorbance of the drug bound to the gelatin was followed and given in Supplementary Table 1. According to the literature, it is understood that free carboplatin in aqueous medium gives a maximum absorbance of 285-310 nm. Therefore, an absorbance peak at 320 nm was observed for only carboplatin, which was not bound to gelatin in the medium.

**Supplementary Table 1**

| Drug | Absorbance | Drug Concentration |
| --- | --- | --- |
| Only Car Pt | 0.1358 | 1 mg/mL |
| 30 °C | 0.1113 | 0.820 mg/mL |
| 35 °C | 0.1002 | 0.738 mg/mL |
| 40 °C | 0.1000 | 0.737 mg/mL |
| 45 °C | 0.0977 | 0.720 mg/mL |
| 50 °C | 0.0935 | 0.689 mg/mL |

**EDX Analysis**

**
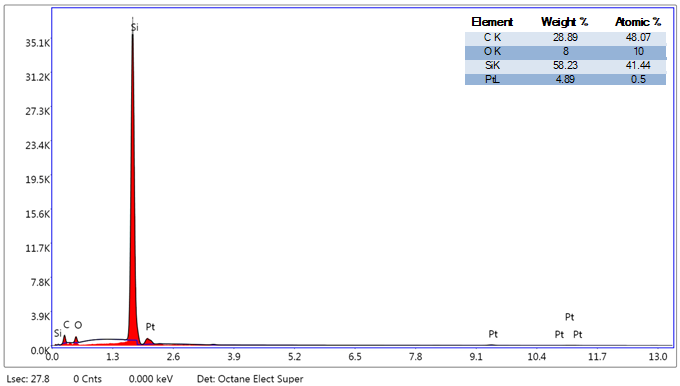
**

**Supplementary Figure 3 (a) 30 °C**

**
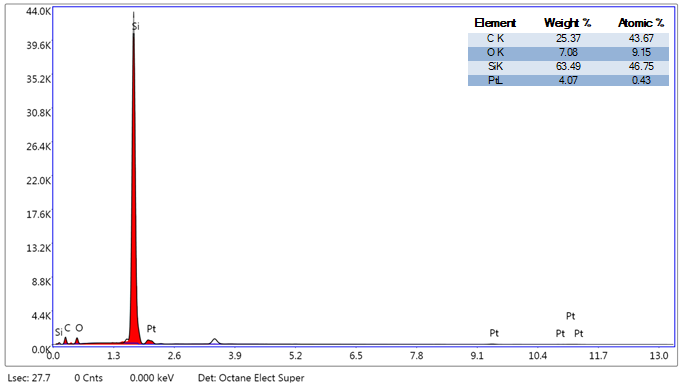
**

**Supplementary Figure 3 (b) 35 °C**

**
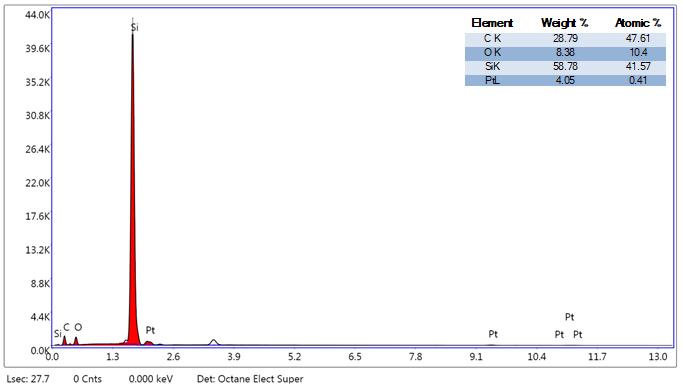
**

**Supplementary Figure 3 (c) 40 °C**

**
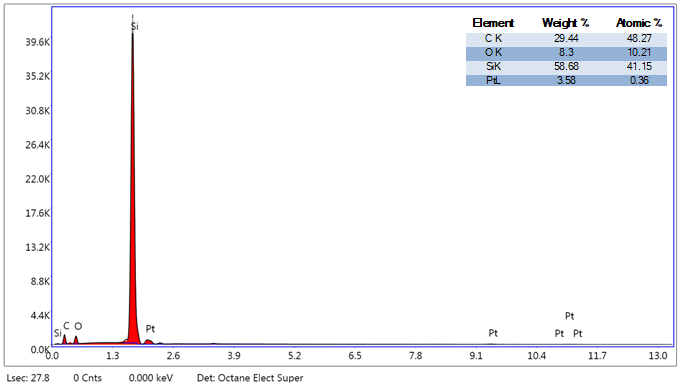
**

**Supplementary Figure 3 (d) 45 °C**

**
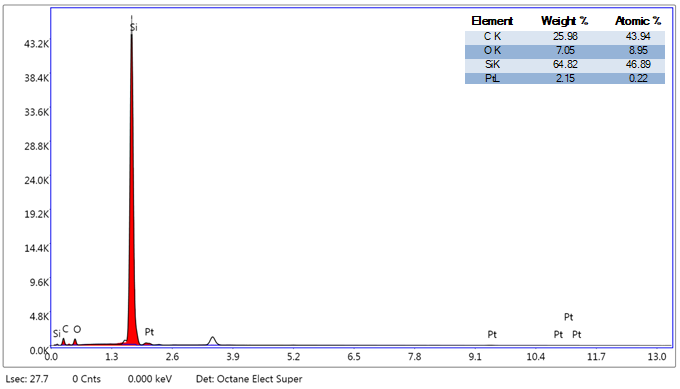
**

**Supplementary Figure 3 (e) 50 °C**

1. Corresponding Author: İshak Afşin Kariper

   e-mail: akariper@erciyes.edu.tr [↑](#footnote-ref-1)
